# Impact of visceral fat on gene expression profile in peripheral blood cells in obese Japanese subjects

**DOI:** 10.1186/s12933-016-0479-1

**Published:** 2016-11-29

**Authors:** Yoshinari Obata, Norikazu Maeda, Yuya Yamada, Koji Yamamoto, Seiji Nakamura, Masaya Yamaoka, Yoshimitsu Tanaka, Shigeki Masuda, Hirofumi Nagao, Shiro Fukuda, Yuya Fujishima, Shunbun Kita, Hitoshi Nishizawa, Tohru Funahashi, Ken-ichi Matsubara, Yuji Matsuzawa, Iichiro Shimomura

**Affiliations:** 1 Department of Metabolic Medicine, Graduate School of Medicine, Osaka University, 2-2-B5 Yamada-oka, Suita, Osaka 565-0871 Japan; 2 Department of Metabolism and Atherosclerosis, Graduate School of Medicine, Osaka University, 2-2-B5 Yamada-oka, Suita, Osaka 565-0871 Japan; 3 Department of Endocrinology and Metabolism, Sumitomo Hospital, 5-3-20, Nakanoshima, Kita-ku, Osaka, Osaka 530-0005 Japan; 4 DNA Chip Research Inc., 1-15-1 Kaigan, Suzuebaydium 5F, Minato-ku, Tokyo, 105-0022 Japan

**Keywords:** Obesity, Visceral fat, Subcutaneous fat, Fat distribution, Gene expression, Microarray, Metabolic syndrome, Diabetes, Adiponectin, KLF

## Abstract

**Background:**

Visceral fat plays a central role in the development of metabolic syndrome and atherosclerotic cardiovascular diseases. The association of visceral fat accumulation with cardio-metabolic diseases has been reported, but the impact of visceral fat on the gene expression profile in peripheral blood cells remains to be determined. The aim of this study was to determine the effects of visceral fat area (VFA) and subcutaneous fat area (SFA) on the gene expression profile in peripheral blood cells of obese subjects.

**Methods:**

All 17 enrolled subjects were hospitalized to receive diet therapy for obesity (defined as body mass index, BMI, greater than 25 kg/m^2^). VFA and SFA were measured at the umbilical level by computed tomography (CT). Blood samples were subjected to gene expression profile analysis by using SurePrint G3 Human GE Microarray 8 × 60 k ver. 2.0. The correlation between various clinical parameters, including VFA and SFA, and peripheral blood gene expression levels was analyzed.

**Results:**

Among the 17 subjects, 12 had normal glucose tolerance or borderline diabetes, and 5 were diagnosed with type 2 diabetes without medications [glycated hemoglobin (HbA1c); 6.3 ± 1.3%]. The mean BMI, VFA, and SFA were 30.0 ± 5.5 kg/m^2^, 177 ± 67 and 245 ± 131 cm^2^, respectively. Interestingly, VFA altered the expression of 1354 genes, including up-regulation of 307 and down-regulation of 1047, under the statistical environment that the parametric false discovery rate (FDR) was less than 0.1. However, no significant effects were noted for SFA or BMI. Gene ontology analysis showed higher prevalence of VFA-associated genes than that of SFA-associated genes, among the genes associated with inflammation, oxidative stress, immune response, lipid metabolism, and glucose metabolism.

**Conclusions:**

Accumulation of visceral fat, but not subcutaneous fat, has a significant impact on the gene expression profile in peripheral blood cells in obese Japanese subjects.

## Background

Increasing evidence demonstrates that excess visceral fat locates upstream of the metabolic syndrome, a cluster of diabetes, dyslipidemia, and hypertension, which is associated with atherosclerotic cardiovascular diseases [1]. In a series of clinical studies, we have shown that visceral fat area (VFA), but not subcutaneous fat area (SFA), correlates significantly and strongly with cardio-metabolic diseases [2, 3]. Various groups, including ours, have focused on the underlying molecular mechanism and links between visceral fat accumulation and cardio-metabolic diseases [4, 5]. Some of the discussed molecular pathological links between visceral adiposity and cardio-metabolic diseases include dysregulation of adipocytokines [1], chronic low-grade inflammation of visceral fat tissue [6], and harmful changes in gut microbiota [7]. However, the exact mechanism(s) remains unresolved.

We have also examined the role of gene expression profile in peripheral blood cells, and reported that visceral adiposity can alter the expression profiles of various genes in peripheral blood cells, including those involved in circadian rhythm and inflammation [8, 9]. However, in these studies, visceral adiposity, including VFA and SFA, was not assessed by modern precision technology such as computed tomography (CT). In addition, impact of SFA on gene expressions in peripheral blood cells was not determined. Moreover, most of the enrolled subjects were overt type 2 diabetes patients (HbA1c; 8.1 ± 2.2%) in our previous study [8, 9], suggesting that gene expression profile in peripheral blood cells influenced by these parameters. Other groups also investigated the impact of VFA and/or SFA on the expression of various genes in peripheral blood cells. For example, Lee et al. [10] found a significant association between VFA, but not SFA, and sirtuin 1 (SIRT1) mRNA level in peripheral blood mononuclear cells.

The aim of the present study was to define the association of VFA and SFA determined by CT, with the gene expression profile in peripheral blood cells in obese subjects free of overt diabetes.

## Methods

### Study population

The enrolled subjects were hospitalized at Sumitomo Hospital between February 2012 and April 2014 to receive calorie-restricted diet therapy for obesity. Subjects with type 1 diabetes mellitus, cancer, autoimmune diseases, and infectious diseases were excluded from the present study. Patients treated with glucose-lowering agents were also excluded. Written informed consent was obtained from each patient after explaining the purpose of study. The study protocol was approved by the human ethics committees of Sumitomo Hospital and Osaka University. The study was also registered with the University Hospital Medical Information Network (UMIN #000001663).

### Clinical parameters

Obesity was defined as body mass index (BMI) greater than 25 kg/m^2^ according to the criteria of the Japan Society for the Study of Obesity [11]. VFA and SFA were measured on the cross-sectional CT slice at the umbilical level [12]. Waist circumference was measured with a tape at the umbilical level in standing position. Serum adiponectin concentration was measured by a latex particle-enhanced turbidimetric immunoassay with a human adiponectin latex kit (Otsuka Pharmaceutical Co., Tokyo, Japan). The homeostasis model − assessment of insulin resistance (HOMA-IR) was calculated by the equation: [HOMA-IR = fasting insulin (µU/mL) × fasting glucose (mg/dL)/405]. Type 2 diabetes mellitus and borderline diabetes were defined according to the criteria of the Japan Diabetes Society [13]. Briefly, diabetes was defined as fasting glucose of ≥126 mg/dL, casual glucose of ≥200 mg/dL, or HbA1c of ≥6.5%. Hypertension was defined as systolic blood pressure (SBP) of ≥140 mm Hg, diastolic BP (DBP) of ≥90 mm Hg, or treatment with anti-hypertensive agents. Dyslipidemia was defined as fasting triglycerides (TG) of ≥150 mg/dL, high-density lipoprotein cholesterol (HDL-C) of <40 mg/dL, or low-density lipoprotein cholesterol (LDL-C) of ≥140 mg/dL, or treatment with lipid-lowering agents. LDL-C was calculated using the Friedewald formula, except in cases with TG of >400 mg/dL. The estimated glomerular filtration rate (eGFR) was calculated by using the following formula: [eGFR = 194 × (serum creatinine^−1.094^) × (age^−0.287^) × F (male, F = 1; female, F = 0.739)] [14]. Intima-media thickness (IMT) of common carotid artery was measured by echography (HI VISION Preirus; Hitachi, Tokyo).

### Microarray analysis

Blood samples were collected into PaxGene Blood RNA tubes (PreAnalytiX, Qiagen Inc., Valencia, CA) before breakfast and left to stand for 2 h at room temperature. The tubes were kept at −20 °C for 2 days and then stored at −80 °C. Total RNA was extracted from the blood sample by using PaxGene Blood RNA Kit (PreAnalytiX, Qiagen). After RNA was qualified by Agilent 2100 Bioanalyzer, 100 ng of total RNA was converted to cDNA, amplified, and labeled with Cy3-labeled CTP using the Quick Amp Labeling kit (Agilent Technologies, Santa Clara, CA). The amplified cRNA and dye incorporation were quantified using ND-1000 Spectrophotometer (Nano Drop Technologies, Wilmington, DE) and hybridized to SurePrint G3 Human GE Microarray 8 × 60 k ver. 2.0 (Design ID: 039494, Agilent Technologies). After hybridization, arrays were washed consecutively by using Gene Expression Wash Pack (Agilent Technologies). Fluorescence images of the hybridized arrays were generated using the Agilent DNA Microarray Scanner, and the intensities were extracted with Agilent Feature Extraction software ver. 10.7.3.1. The raw microarray data are deposited in the National Center for Biotechnology Information Gene Expression Omnibus (GEO Series GSE85226).

### Microarray data analyses

The raw microarray intensities were processed by the percentile shift method (75th percentile) with GeneSpring GX 13.0 (Agilent Technologies) so as to normalize the range of expression intensities for inter-microarray. Genes found to be expressed in more than 50% of the hybridizations were subjected to further analyses. The normalized data were exported from the GeneSpring GX software. The univariate correlation between clinical parameters, including VFA and SFA, and peripheral blood gene expression levels was examined by Pearson’s correlation under the R environment (http://cran.at.r-project.org). Gene ontology (GO) information was retrieved from the annotations in GeneSpring GX 13.0.

## Results

### Characteristics of the enrolled subjects

The clinical characteristics of the participating subjects are listed in Table 1. The mean BMI and waist circumference were 30.0 kg/m^2^ (range, 24.0–44.0 kg/m^2^) and 101.2 cm (range, 85–127 cm), respectively. The mean VFA and SFA were 177.3 cm^2^ (range, 78–318 cm^2^) and 244.7 cm^2^ (range, 80–558 cm^2^), respectively. The mean serum adiponectin concentration was 4.2 μg/mL (range, 2.3–9.8 μg/mL) and the mean HbA1c was 6.3% (range, 5.3–10.9%). Among the 17 subjects, 5 had type 2 diabetes, 6 had borderline diabetes, and 6 subjects had normal glucose tolerance. All 5 diabetic patients were not treated with any anti-diabetic agents. Atherosclerotic plaque in the carotid artery (IMT ≥1.1 mm) was observed in 7 subjects. Among the 17 subjects, dyslipidemia and hypertension were found in 15 and 8 subjects, respectively. Seven patients were treated with statins and four patients were treated with angiotensin converting enzyme inhibitor (ACE-I) or angiotensin II receptor blocker (ARB).

**Table 1:** Characteristics of subjects

N	17
Sex (male/female)	14/3
Age (years)	54.6 ± 14.6
BMI (kg/m^2^)	30 ± 5.5
Waist circumference (cm)	101 ± 11
Visceral fat area (cm^2^)	177 ± 67
Subcutaneous fat area (cm^2^)	245 ± 131
Adiponectin (μg/mL)	4.2 ± 1.7
Systolic blood pressure (mm Hg)	132 ± 17
Diastolic blood pressure (mm Hg)	82 ± 13.7
Fast plasma glucose (mg/dL)	102 ± 21
Hemoglobin A1c (%)	6.3 ± 1.3
Diagnosis (T2DM/B/N)	5/6/6
HOMA-IR	3.2 ± 2.3
Total cholesterol (mg/dL)	206 ± 40
Triglyceride (mg/dL)	196 ± 129
HDL-C (mg/dL)	56.3 ± 18.1
LDL-C (mg/dL)	114 ± 40
Uric acid (mg/dL)	6.4 ± 0.8
Urinary albumin (μg/day)	12.3 ± 10.6
eGFR (mL/min/1.73 m^2^)	77.9 ± 19.8
mean IMT ≥1.1 mm	7/10
Statin use (±)	7/10
ACE-I/ARB use (±)	4/13

Data are mean ± SD

*T2DM* type 2 diabetes mellitus, *B* borderline diabetes, *N* normal glucose tolerance, *HOMA*-*IR* homeostasis model assessment of insulin resistance, *HDL*-*C* high density lipoprotein-cholesterol, *LDL*-*C* low density lipoprotein-cholesterol, *eGFR* estimated glomerular filtration rate, *IMT* intima-media thickness, *ACE*-*I* angiotensin converting enzyme inhibitor, *ARB* angiotensin II receptor blocker

### Gene expression profiles

**Fig. 1:**
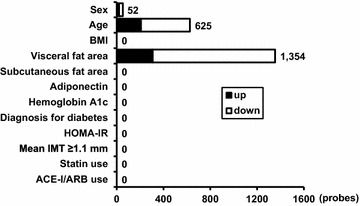
Changes in the number of genes according to various clinical parameters. The target 23,197 probes were selected under the condition that significant signals were detected in more than 7 cases among 17 subjects. Data represent the number of probes that showed significant upregulation and downregulation according to the listed clinical parameters under the statistical environment that the parametric false discovery rate (FDR) was less than 0.1. Parameters such as sex, diagnosis for diabetes, mean IMT, statin use, and ACE-I/ARB use were adopted as categorical variables. Age, BMI, visceral and subcutaneous fat areas, adiponectin, hemoglobin A1c, and HOMA-IR were adopted as continuous variables. *BMI* body mass index; *HOMA-IR* homeostasis model assessment of insulin resistance; *IMT* intima-media thickness; *ACE-I* angiotensin converting enzyme inhibitor; *ARB* angiotensin II receptor blocker

Peripheral blood RNA samples were subjected to microarray analysis. The target probes were selected under the condition that significant signals were detected in more than 7 cases among 17 subjects and thus 23,197 probes were extracted for gene expression analysis. Table 2 lists the number of probes that showed significant changes according to various clinical variables under the statistical environment that the parametric false discovery rate (FDR) was less than 0.1. Sex and age had impacts on 52 and 625 probes, respectively. Surprisingly, VFA had a great impact on peripheral blood cells gene expression, i.e., 1354 probes consisting of 307 up-regulated and 1047 down-regulated probes. However, no significant gene probes were detected with SFA or BMI. Serum adiponectin, diabetes, HbA1c, and HOMA-IR also had no impact on the gene expression in peripheral blood cells. Likewise, statins and ACE-I/ARB had no effect. Figure 1 illustrates the number of upregulated/downregulated probes according to various clinical parameters. Table 3 lists the top 30 genes that correlated significantly with VFA positively and negatively. Among these genes, Krüppel-like factor 10 (KLF10) was the most significant (Table 3).

**Table 2:** Changes in probes according to various clinical parameters

	FDR < 0.1	Up	Down
*Categorical*			
Sex	52	20	32
Diagnosis of diabetes	0	0	0
Mean IMT	0	0	0
Statin use	0	0	0
ACE-I/ARB use	0	0	0
*Continuous*			
Age	625	206	419
Body mass index	0	0	0
Visceral fat area	1354	307	1047
Subcutaneous fat area	0	0	0
Adiponectin	0	0	0
Hemoglobin A1c	0	0	0
HOMA-IR	0	0	0

Data represent number of probes

*FDR* false discovery rate, *IMT* intima-media thickness, *ACE*-*I* angiotensin converting enzyme inhibitor, *ARB* angiotensin II receptor blocker, *HOMA*-*IR* homeostasis model assessment of insulin resistance

**Table 3:** Top 30 genes that correlated positively and negatively with visceral fat area

Probe name	Gene symbol	Gene name	R	*p* value	FDR
*Positive correlation*					
A_21_P0013668	SPATA31C2	SPATA31 subfamily C, member 2	0.830	3.72E−05	0.08430563
A_19_P00803850	LOC100505474	Uncharacterized LOC100505474	0.828	4.01E−05	0.08430563
A_33_P3238410	SBF1	SET binding factor 1	0.814	6.93E−05	0.08430563
A_23_P325676	ZNF653	Zinc finger protein 653	0.803	1.03E−04	0.08430563
A_23_P384532	CCDC11	Coiled-coil domain containing 11	0.802	1.07E−04	0.08430563
A_33_P3311956	FEZ2	Fasciculation and elongation protein zeta 2 (zygin II)	0.801	1.13E−04	0.08430563
A_23_P430670	CHST5	Carbohydrate (N-acetylglucosamine 6-O) sulfotransferase 5	0.797	1.29E−04	0.08430563
A_33_P3253653	GPR155	G protein-coupled receptor 155	0.779	2.29E−04	0.08430563
A_33_P3314974	PARD6G-AS1	PARD6G antisense RNA 1	0.778	2.33E−04	0.08430563
A_33_P3402773			0.775	2.61E−04	0.084956675
A_24_P117942	TOMM20L	Translocase of outer mitochondrial membrane 20 homolog (yeast)-like	0.771	2.94E−04	0.084956675
A_33_P3772937	KRT8P12	Keratin 8 pseudogene 12	0.768	3.15E−04	0.084956675
A_33_P3404889			0.768	3.20E−04	0.084956675
A_33_P3379436	FAM74A4	Family with sequence similarity 74, member A4	0.767	3.29E−04	0.084956675
A_23_P34066	IL9R	Interleukin 9 receptor	0.765	3.50E−04	0.084956675
A_23_P417415	ACOT11	Acyl-CoA thioesterase 11	0.758	4.19E−04	0.084956675
A_33_P3410093	LTA4H	Leukotriene A4 hydrolase	0.758	4.22E−04	0.084956675
A_33_P3334895	GRIN2A	Glutamate receptor, ionotropic, N-methyl D-aspartate 2A	0.750	5.26E−04	0.084956675
A_23_P72697	GPIHBP1	Glycosylphosphatidylinositol anchored high density lipoprotein binding protein 1	0.749	5.40E−04	0.084956675
A_33_P3378531	AS3MT	Arsenite methyltransferase	0.748	5.57E−04	0.084956675
A_24_P75190	HBD	Hemoglobin, delta	0.747	5.76E−04	0.084956675
A_23_P26457	HBA2	Hemoglobin, alpha 2	0.744	6.08E−04	0.084956675
A_33_P3265866			0.744	6.09E−04	0.084956675
A_21_P0004859	BTN2A1	Butyrophilin, subfamily 2, member A1	0.741	6.63E−04	0.084956675
A_21_P0005185	DKFZp686L13185	Uncharacterized LOC401287	0.739	6.95E−04	0.084956675
A_21_P0012204	XLOC_014512		0.739	7.00E−04	0.084956675
A_21_P0009476	XLOC_012670		0.737	7.45E−04	0.084956675
A_33_P3365932	WASH1	WAS protein family homolog 1	0.734	7.91E−04	0.084956675
A_19_P00812257	LINC01191	Long intergenic non-protein coding RNA 1191	0.732	8.26E−04	0.084956675
A_23_P209564	CYBRD1	Cytochrome b reductase 1	0.732	8.36E−04	0.084956675
*Negative correlation*					
A_23_P168828	KLF10	KRUPPEL-like factor 10	−0.856	1.16E−05	0.08430563
A_32_P54544	CCT6A	Chaperonin containing TCP1, subunit 6A (zeta 1)	−0.832	3.40E−05	0.08430563
A_23_P389919	WHSC1	Wolf-Hirschhorn syndrome candidate 1	−0.830	3.74E−05	0.08430563
A_23_P44139	PRIM2	Primase, DNA, polypeptide 2 (58 kDa)	−0.827	4.21E−05	0.08430563
A_19_P00331853	LOC100131564	uncharacterized LOC100131564	−0.826	4.34E−05	0.08430563
A_23_P501877	ZFP64	ZFP64 zinc finger protein	−0.826	4.36E−05	0.08430563
A_24_P3973	HNRNPA2B1	Heterogeneous nuclear ribonucleoprotein A2/B1	−0.826	4.46E−05	0.08430563
A_23_P215088	ZC3HC1	Zinc finger, C3HC-type containing 1	−0.826	4.47E−05	0.08430563
A_23_P7679	NUP155	Nucleoporin 155 kDa	−0.816	6.50E−05	0.08430563
A_33_P3381483	ZNF331	Zinc finger protein 331	−0.811	7.73E−05	0.08430563
A_23_P115149	WDR77	WD repeat domain 77	−0.808	8.71E−05	0.08430563
A_23_P151093	YARS2	Tyrosyl-tRNA synthetase 2, mitochondrial	−0.807	8.92E−05	0.08430563
A_23_P251421	CDCA7	Cell division cycle associated 7	−0.807	8.94E−05	0.08430563
A_21_P0008290	LINC00641	Long intergenic non-protein coding RNA 641	−0.807	9.17E−05	0.08430563
A_33_P3262665	MAP7D3	MAP7 domain containing 3	−0.805	9.59E−05	0.08430563
A_33_P3213557	CCZ1	CCZ1 vacuolar protein trafficking and biogenesis associated homolog (*S. cerevisiae*)	−0.805	9.60E−05	0.08430563
A_23_P202143	NOLC1	Nucleolar and coiled-body phosphoprotein 1	−0.804	1.01E−04	0.08430563
A_23_P46924	BUB3	BUB3 mitotic checkpoint protein	−0.803	1.04E−04	0.08430563
A_24_P925635	SEPT7P2	Septin 7 pseudogene 2	−0.803	1.06E−04	0.08430563
A_24_P345822	TFG	TRK-fused gene	−0.802	1.08E−04	0.08430563
A_23_P85180	TMEM187	Transmembrane protein 187	−0.801	1.10E−04	0.08430563
A_33_P3221234	IPP	Intracisternal A particle-promoted polypeptide	−0.801	1.13E−04	0.08430563
A_33_P3415037	VDAC2	Voltage-dependent anion channel 2	−0.799	1.20E−04	0.08430563
A_33_P3309929	HDAC3	Histone deacetylase 3	−0.799	1.21E−04	0.08430563
A_23_P214798	SYNCRIP	Synaptotagmin binding, cytoplasmic RNA interacting protein	−0.796	1.34E−04	0.08430563
A_21_P0012709	XLOC_014512		−0.795	1.38E−04	0.08430563
A_24_P116909	MALT1	Mucosa associated lymphoid tissue lymphoma translocation gene 1	−0.794	1.42E−04	0.08430563
A_23_P69437	YEATS2	YEATS domain containing 2	−0.793	1.45E−04	0.08430563
A_33_P3251538	MAPKAP1	Mitogen-activated protein kinase associated protein 1	−0.793	1.46E−04	0.08430563
A_23_P102202	MSH6	Muts homolog 6	−0.793	1.47E−04	0.08430563

### Gene ontology

**Fig. 2:**
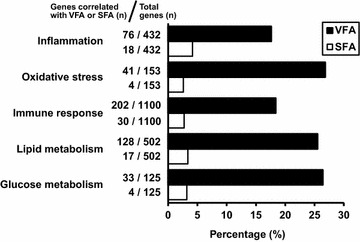
Percentages of obesity-related genes that correlated significantly with visceral and subcutaneous fat area. Gene ontology analysis was performed to examine the impact of visceral fat area (VFA) and subcutaneous fat area (SFA) on the percentage of obesity-associated genes (relative to total number of genes), such as inflammation (GO:0006954), oxidative stress (GO:0006979), immune response (GO:0006955), lipid metabolism (GO:0006629), and glucose metabolism (GO:0006006)

Gene ontology (GO) analysis was also performed to further determine the impact of VFA on gene expression profile in peripheral blood cells. As shown in Table 4, visceral fat adiposity correlated significantly with genes related to the metabolic process, oxygen transport, and nucleotide binding. Genes involved in inflammation (GO: 0006954), oxidative stress (GO: 0006979), immune response (GO: 0006955), lipid metabolism (GO: 0006629), and glucose metabolism (GO: 0006006), were finally examined. Figure 2 shows the percentage of genes (among all genes) that correlated significantly with SFA and VFA (*p* < 0.05). VFA correlated with 17.6, 26.8, 18.4, 25.5, and 26.4% of genes involved in inflammation, oxidative stress, immune response, lipid metabolism, and glucose metabolism, respectively, while the respective percentages for SFA were only 4.2, 2.6, 2.7, 3.4, and 3.2%.

**Table 4:** Significant GO terms based on genes that correlated positively and negatively with visceral fat area

GO	GO term	Corrected *p* value
*Positive correlation*		
Biological process	Oxygen transport	8.821E−04
	Gas transport	3.707E−03
Molecular function	Oxygen transporter activity	5.310E−04
Cellular component	Hemoglobin complex	3.428E−04
*Negative correlation*		
Biological process	RNA processing	6.559E−21
	Heterocycle metabolic process	1.252E−20
	Cellular nitrogen compound metabolic process	8.348E−20
	Nucleobase-containing compound metabolic process	1.702E−19
	Organic cyclic compound metabolic process	2.892E−19
	Cellular aromatic compound metabolic process	6.642E−19
	Cellular metabolic process	4.940E−18
	Nitrogen compound metabolic process	9.293E−18
	Nucleic acid metabolic process	1.052E−16
	Cellular macromolecule metabolic process	1.780E−16
	Metabolic process	1.122E−14
	Primary metabolic process	1.551E−14
	Gene expression	3.068E−14
	Organic substance metabolic process	7.768E−14
	Macromolecule metabolic process	1.529E−11
	RNA metabolic process	3.440E−11
	mRNA processing	5.194E−11
	ncRNA metabolic process	1.859E−10
	RNA splicing	5.494E−10
Molecular function	RNA binding	1.470E−10
	Nucleotide binding	2.106E−07
	Nucleoside phosphate binding	2.143E−07
	Heterocyclic compound binding	1.144E−06
	Nucleic acid binding	2.081E−06
	Aminoacyl-tRNA ligase activity	2.924E−06
	Ligase activity, forming aminoacyl-tRNA and related compounds	2.924E−06
	Ligase activity, forming carbon–oxygen bonds	2.924E−06
	Organic cyclic compound binding	3.035E−06
	Small molecule binding	3.265E−06
	Catalytic activity	1.736E−04
	ATP-dependent helicase activity	3.046E−04
	Purine NTP-dependent helicase activity	3.046E−04
	Structure-specific DNA binding	3.201E−04
	ATPase activity	9.197E−04
	ATPase activity, coupled	1.738E−03
	Adenyl nucleotide binding	1.738E−03
	ATP binding	1.799E−03
	Adenyl ribonucleotide binding	2.557E−03
Cellular component	Nuclear part	2.984E−28
	Intracellular part	6.115E−28
	Intracellular	1.984E−27
	Intracellular membrane-bounded organelle	8.017E−27
	Membrane-enclosed lumen	6.105E−24
	Intracellular organelle lumen	6.201E−24
	Nuclear lumen	6.172E−23
	Organelle lumen	8.194E−23
	Intracellular organelle part	2.042E−22
	Intracellular organelle	2.087E−22
	Membrane-bounded organelle	1.535E−21
	Organelle part	3.743E−21
	Nucleus	1.105E−20
	Organelle	1.383E−17
	Nucleolus	2.194E−16
	Nucleoplasm	4.535E−15
	Mitochondrion	1.779E−13
	Mitochondrial part	1.863E−09
	Cytoplasm	2.197E−09

## Discussion

The major finding of the present study was that visceral fat, but not subcutaneous fat, in obese individuals had a significant impact on peripheral blood cells gene expression profile. While similar results were reported previously by our group [8, 9], these studies had several limitations: (1) VFA was estimated by abdominal bioelectrical impedance analysis (BIA), rather than by CT. The latter is recognized as the gold standard method for fat area measurement [12, 15, 16]. (2) The majority of the subjects enrolled in the above previous studies were diabetics (75%) with a mean HbA1c of 8.1%. The inclusion of such patients could have influenced the results. (3) Impact of SFA on gene expression level in peripheral blood cells could not be determined under abdominal BIA procedure. The present study is clinically more significant as it included precise measurement of VFA and SFA by CT scan and negligible diabetic conditions.

The biological differences between visceral and subcutaneous fat have been investigated. The rate of lipolysis and lipogenesis activities are higher in adipocytes of visceral fat tissue than those of subcutaneous fat tissue [17, 18], suggesting that visceral fat accumulation increases free fatty acids (FFA) in the portal vein, accelerates hepatic lipogenesis, and results in dyslipidemia involving high FFA level in the bloodstream. Visceral fat accumulation also enhances inflow of glycerol into the liver and hepatic glucose production through adipose and hepatic glycerol channels; aquaporin 7 and 9, respectively [19]. Furthermore, adipose mRNA levels dynamically change in visceral fat compared to subcutaneous fat, especially in obese subjects. As BMI increases, the mRNA levels of adiponectin and peroxisome proliferator-activated receptor gamma (PPARγ) are reduced, while mRNA level of NADPH oxidase subunit p22, promoting reactive oxygen species (ROS), is augmented, in visceral fat, but not in subcutaneous fat [20]. Visceral fat accumulation is also a major risk for the reduction of circulating adiponectin (hypoadiponectinemia) [1]. Collectively, compared to subcutaneous fat, visceral fat accumulation largely and pathologically alters not only its own fat tissue, but also circulating substances and metabolic outcome. It is therefore conceivable that these visceral fat-mediated changes can also alter the gene expression profile in peripheral blood cells.

Increasing evidence indicates that chronic low-grade inflammation in the adipose tissue, especially in visceral fat, is located upstream of the metabolic syndrome [21, 22]. Gut microbiota also accelerates inflammatory changes in visceral fat [7]. Various immune cells infiltrate adipose tissue and cause inflammatory changes through direct cell–cell interaction and/or indirect cytokine-mediated intercellular communication. It is not hard to imagine that such interactions among immune cells and adipocytes influence peripheral blood cells, but such processes have not been confirmed yet. The present study also suggests that gene expression profile of peripheral blood cells reflects local inflammatory changes in visceral fat.

Interestingly, KLF10, a member of the Krüppel-like family of transcription factors, showed the most significant and negative correlation with VFA (Table 3). KLF10 is augmented through the transforming growth factor-β (TGF-β)-Smad signaling pathway [23]. It plays a crucial role in TGF-β-mediated induction of regulatory T-cells (Treg) from naive T-cells [24]. In mice lacking KLF10, Treg activity was reduced and proinflammatory changes were accelerated. Transfer of KLF10-deficient T-cells failed to suppress the development of atherosclerosis in apolipoprotein E knockout mice with high-fat diet [25]. KLF10-deficient mice also showed hyperglycemia in males and hypertriglyceridemia in females [26]. KLF10 has been shown to regulate 20–30% of hepatic genes related to glucose and lipid metabolism [26]. Genetic variants of KLF10 are associated with susceptibility to type 2 diabetes [27]. However, KLF10 mRNA expressions were not significantly correlated with diabetes or dyslipidemia in present study. To confirm the association between KLF10 expressions in peripheral blood cells and diabetes or dyslipidemia, further investigations would be desired in some other populations, different from present clinical profiles, such as non-obese or non-diabetic subjects. Present data provides a possibility that visceral fat adiposity-associated reduction in peripheral blood KLF10 mRNA level is related to the pathogenesis of the metabolic syndrome, although further clinical studies would be needed in future.

The present study has several limitations. The study population was small and the proportion of female was low. Several participants received medications such as statins and ACE-I/ARBs. Importantly, the majority of subjects were obese and showed abundant accumulation of visceral fat according to the Japanese criteria; the study included only one subject with VFA below 100 cm^2^. The full impact of VFA on the gene expression profile of peripheral blood cells has not been determined previously and should be examined also in non-obese individuals. In the present study, among the top 30 genes that correlated positively with VFA (Table 3), 14 (46.7%) genes were up-regulated in obesity, and among the top 30 genes that correlated negatively with VFA (Table 3), 17 (56.7%) genes were down-regulated in obesity. Unfortunately, a control group of non-obese subjects could not be included in the present study for ethical reasons (exposure of such subjects to CT scanning). For this reason, no data are available for the correlation of VFA and SFA to the gene expression profile of peripheral blood cells in non-obese subjects. Therefore, our results can only be applied to obese individuals.

## Conclusions

The present study demonstrated that accumulation of visceral fat, but not that of subcutaneous fat, alters the gene expression profile of peripheral blood cells in obese Japanese subjects. The results should enhance our understanding of the pathogenesis of the metabolic syndrome.
